# Prediction of early‐onset colorectal cancer mortality rates in the United States using machine learning

**DOI:** 10.1002/cam4.6880

**Published:** 2023-12-27

**Authors:** Hassam Ali, Pratik Patel, Dushyant Singh Dahiya, Manesh Kumar Gangwani, Debargha Basuli, Babu Pappu Mohan

**Affiliations:** ^1^ Department of Gastroenterology East Carolina University/Brody School of Medicine Greenville North Carolina USA; ^2^ Department of Gastroenterology Mather Hospital/Hofstra University Zucker School of Medicine New York City New York USA; ^3^ Division of Gastroenterology, Hepatology & Motility The University of Kansas School of Medicine Kansas City Kansas USA; ^4^ Department of Medicine University of Toledo Medical Center Toledo Ohio USA; ^5^ Department of Internal Medicine ECU health medical center/Brody School of Medicine Greenville North Carolina USA; ^6^ Orlando Gastroenterology PA Orlando Florida USA

**Keywords:** autoregressive integrated moving average, cancer prevention, colorectal cancer, machine learning, mortality trends

## Abstract

**Introduction:**

The current study, focusing on a significant US (United States) colorectal cancer (CRC) burden, employs machine learning for predicting future rates among young population.

**Methods:**

CDC WONDER data from 1999 to 2022 was analyzed for CRC‐related mortality in patients younger than 56 years. Temporal trends in age‐adjusted mortality rates (AAMRs) were assessed via Joinpoint software. Future mortality rates were forecasted using an optimal Autoregressive Integrated Moving Average (ARIMA) model.

**Results:**

From 1999 to 2022, we observed 150,908 deaths with CRC listed as the underlying cause, predominantly in males, with an upward trend in AAMR. The ARIMA model projects an increase in CRC mortality by 2035, estimating an average annual percent change (AAPC) of 1.3% overall, 1% for females, and 1.5% for males.

**Conclusion:**

Our study findings emphasize the need for more robust preventive measures to reduce future CRC mortality among younger population. These results have significant implications for public health policies, particularly for males under 56, and underscore the importance of early screening and lifestyle modifications.

## 1 INTRODUCTION

Colorectal cancer (CRC) is a significant public health concern in the United States (US), with an estimated 147,950 new cases and 53,200 deaths reported in 2020.^1^ Despite being the third most common cancer and the second leading cause of cancer‐related deaths in the US, recent trends have shown a rise in CRC incidence among individuals aged 55 years or younger.^2^ Advanced CRC is more prevalent in the younger demographic, in part because of screening inconsistencies. Additionally, lifestyle factors such as increased sedentary behavior, poor diet, and genetic predispositions may contribute to this rising trend. The lack of early detection often stems from less frequent screening and misdiagnosis, further exacerbating the issue.

Current research demonstrates that establishing a diagnosis of CRC takes 40% longer in patients under the age of 50, often due to prolonged symptom evaluation and misdiagnosis.^2^ The latest data shows that CRC incidence and mortality rates have decreased overall.^3^, ^4^ The US Preventive Services Task Force (USPSTF) recently updated its recommendation, lowering the age for screening colonoscopy from 50 to 45 years.^5^ This change is expected to lead to earlier CRC detection, thereby reducing the incidence and mortality rates.

Predicting future trends in CRC rates is complex due to factors such as demographic changes, risk factor prevalence, and healthcare practices. In this study, we apply machine learning techniques to predict future trends in CRC‐related mortality rates in adult patients younger than 56 of age in the US.

## 2 METHODS

We retrieved deidentified data from the Centers for Disease Control and Prevention Wide‐Ranging Online Data for Epidemiologic Research (CDC WONDER) multiple causes of death database (years 1999–2022) for CRC‐related mortality with a focus on the underlying cause of death. The study population consisted of patients with CRC as defined by the International Classification of Diseases‐10 codes (C18, C19, and C20).^4^

All statistical analyses were performed using *Python*, utilizing the *PyCharm Integrated Development Environment* (IDE) and *Generative Pre‐trained Transformer 4* (GPT‐4).^6^ Python and GPT‐4 were chosen for their advanced statistical capabilities, which enhanced the validity of our study by allowing for more complex analyses and data visualization. The age‐adjusted mortality rates (AAMRs) per 100,000 population among individuals aged 25–55 were examined. AAMRs were standardized to the 2000 US population. Joinpoint software assessed Annual Average Percent Change (AAPC) temporal trends with 95% confidence intervals (CI) and *p*‐values, representing the change in mortality during a specific period. We chose to focus on the age range of 25–55 years to capture the emerging trend of rising CRC incidence in younger populations. This age range is particularly relevant as it represents a demographic that is increasingly being affected by CRC but is often overlooked in screening programs.

For predictive time series analysis, the *autoregressive integrated moving average* (ARIMA) model was used for non‐stationary data in the context of the long‐term trend to forecast mortality rates till 2035, as previously.^7^ The ARIMA model was selected for its robustness in handling non‐stationary time series data and its widespread use in healthcare forecasting. Compared to other models, ARIMA provides a more nuanced understanding of time‐dependent patterns.^7^, ^8^ An optimal ARIMA model, identified using the auto ARIMA function based on the Bayesian Information Criterion (BIC), was fitted to the data. The model's residuals were evaluated for white noise via the Ljung‐Box test.^8^ The model's robustness was validated using time series cross‐validation (*n* = 10), with Root Mean Squared Error (RMSE) reported for accuracy.^9^

The study adhered to STROBE reporting standards and didn't require informed consent or institutional board approval as it used anonymized public data, following the Common Rule.

## 3 RESULTS

**FIGURE 1:**
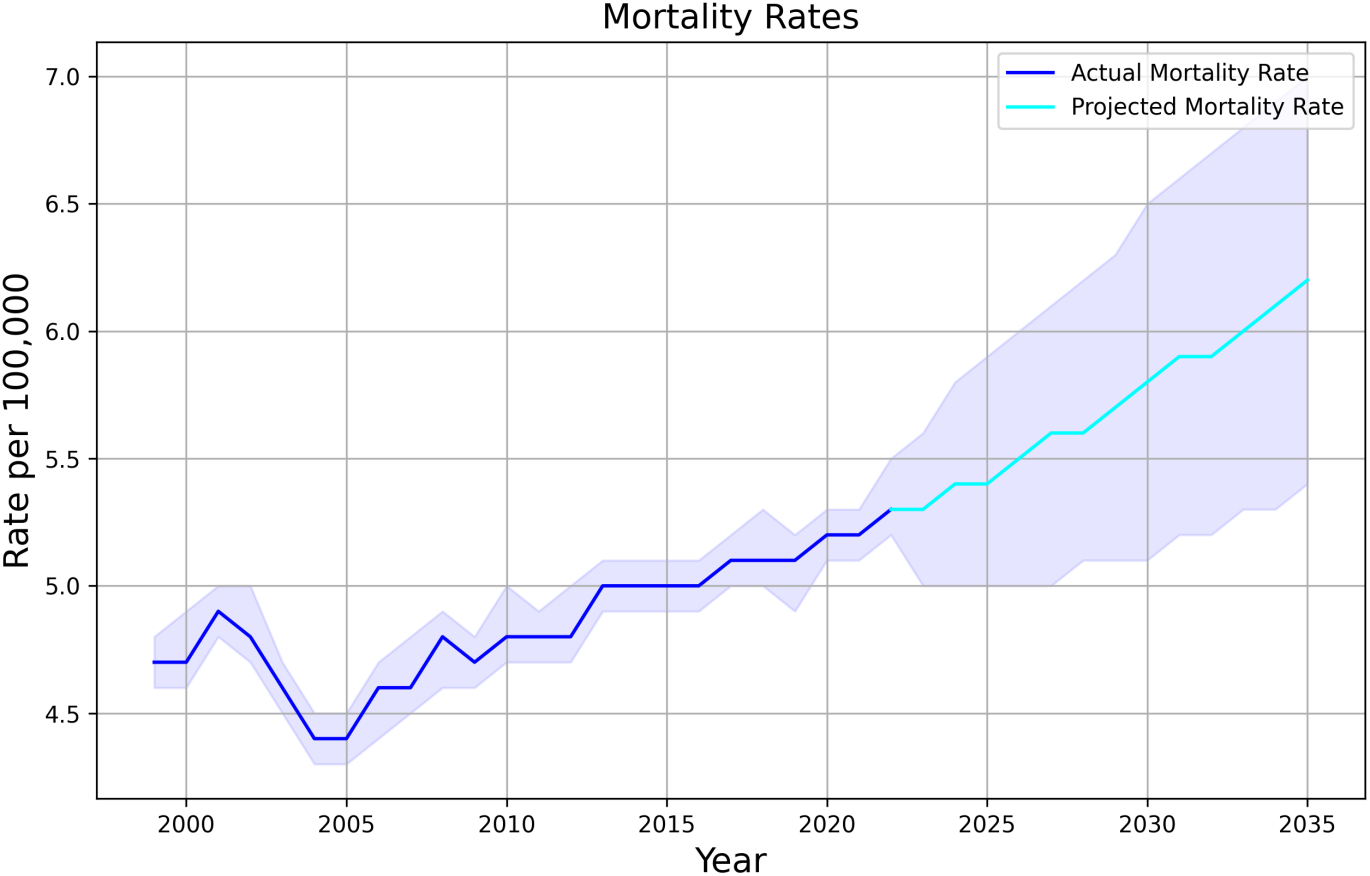
Actual and projected rates of colorectal cancer‐related mortality up to 2035. The shaded areas represent the 95% confidence intervals.

Between 1999 and 2022, there were 150,908 deaths with CRC as the underlying cause of death (males 56.4%). Overall, the AAMR increased from 4.7/100,000 in 1999 to 5.3/100,000 in 2022, showing an AAPC of 0.6% (95% CI 0.4, 0.8, *p* < 0.001) (Figure 1). For females, AAMR increased from 4.2/100,000 in 1999 to 4.6/100,000 in 2022, showing an AAPC of 0.3% (95% CI 0.1, 0.5, *p* = 0.007). For males, AAMR increased from 5.2/100,000 in 1999 to 6.1/100,000 in 2022, showing an AAPC of 0.8% (95% CI 0.5, 1.0, *p* < 0.001).

**FIGURE 2:**
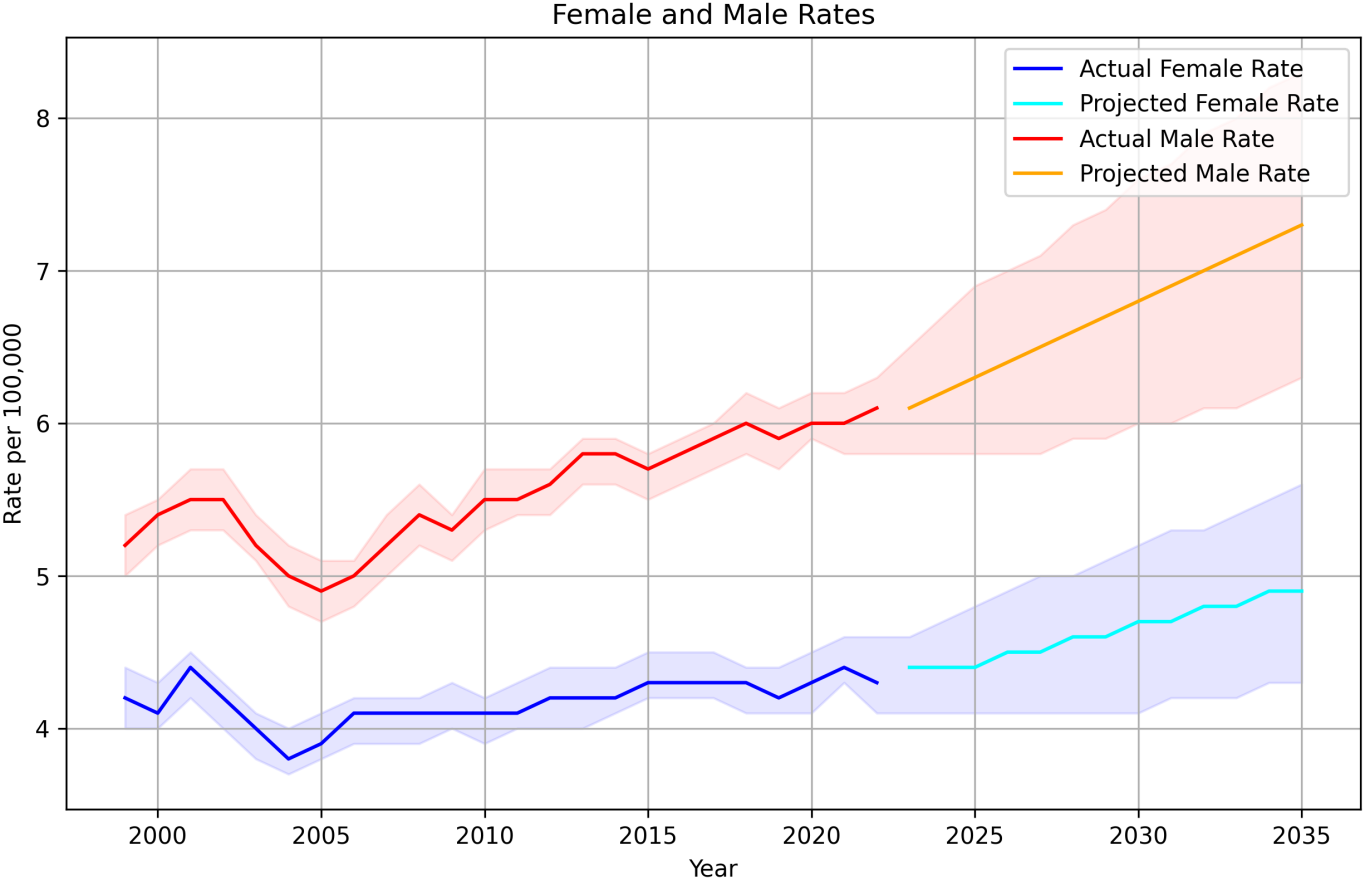
Actual and projected rates of colorectal cancer‐related mortality up to 2035, stratified by gender. The shaded areas represent the 95% confidence intervals.

The optimal ARIMA models were chosen based on the lowest BIC and a satisfactory Ljung‐Box test, indicating that the residuals are independently distributed. This was preferred as it minimized information loss while adequately capturing the underlying trend in the data. The ARIMA model (0, 1, 0) with a BIC of −30.3 was selected for total patients. The Ljung‐Box test indicated that the residuals are independently distributed (*p* = 0.99). The model was cross‐validated using a 10‐fold TimeSeriesSplit, and the average Root Mean Squared Error (RMSE) was 3.15. The model was then used to forecast CRC mortality rates from 2023 to 2035. The forecasted rate for 2023 was 5.31/100,000 (95% CI: 4.99–5.63), with a projected increase to 6.20/100,000 (95% CI: 5.37–7.03) by 2035 with an AAPC of 1.3% (95% CI 1.2, 1.4, *p* < 0.001) (Figure 1). For females, the forecasted rate for 2023 was 4.36/100,000 (95% CI: 4.07–4.64), with a projected increase to 4.92/100,000 (95% CI: 4.2–5.55) by 2035 with an AAPC of 1% (95% CI 0.9, 1.1, *p* < 0.001; ARIMA model (0, 1, 0) with a BIC of −25.5, RMSE = 0.14). For males, the forecasted rate for 2023 was 6.15/100,000 (95% CI: 5.76–6.53), with a projected increase to 7.31/100,000 (95% CI: 6.30–8.33) by 2035 with an AAPC of 1.5% (95% CI 1.4, 1.5, *p* < 0.001; ARIMA model [0, 1, 0] with a BIC of −21.1, RMSE = 1.18; Figure 2).

## 4 DISCUSSION

This study highlights an upward trend in CRC mortality rates between 1999 and 2022, with our model predicting a continued increase through 2035. The rise in AAMR is more pronounced in males, suggesting the necessity of gender‐focused preventive and therapeutic strategies. The increasing trend, up to 2035, was similar when the 2015–2022 data was used, however, with a higher error factor. Therefore, it can be safely deduced that current CRC screening outreach must be widened to achieve a downward trend in CRC‐related mortality through 2035. Compared to existing literature that primarily focuses on the epidemiology of CRC, our study adds a new dimension by employing machine learning techniques for predictive modeling.^10^ This is a significant strength of our study as it provides actionable insights for public health policies.

An important aspect to consider in the context of early‐onset colorectal cancer is the role of rectum cancer. Literature has indicated that rectum cancer is a significant contributor to early‐onset CRC, and its inclusion in the analysis is crucial for a comprehensive understanding of the disease.^11^ While our study specifically looks at colorectal cancer, future studies are needed to investigate the prediction of both cancers separately to provide a more nuanced understanding of early‐onset colon and rectal cancers.

While the literature has focused on the epidemiology and biology of early‐onset CRC to the best of our knowledge,^12^ there are limited studies specifically aimed at predicting early‐onset CRC. This makes our study unique and adds strength to our findings. However, it is important to note that risk factors for early‐onset CRC have been well‐studied, including increasing age, higher BMI or obesity, and family history of cancer.^13^ Our study aligns with these findings, extends them by providing predictive models for future trends, and underscores the urgent need for interventions targeting these modifiable risk factors, as highlighted in existing literature.

The acceleration in mortality rates projected by our models could be associated with increased CRC risk factors such as a sedentary lifestyle, obesity, and poor diet. This highlights the importance of robust preventive measures, including regular screening, lifestyle modifications, and effective treatment strategies. In addition to increasing awareness of screening modalities, achieving compliance needs to be addressed.

The observed gender disparities in CRC mortality rates may be influenced by a variety of factors. Men may be exposed to different risk factors, such as occupational hazards or lifestyle choices, compared to women. Additionally, access to healthcare and biological differences could play a role in these disparities. Our findings align with previous studies that have also reported gender disparities in CRC mortality rates, but we extend this by providing future projections that can guide gender‐specific interventions.^14^ It's worth noting that future changes in CRC prevention and treatment, such as advancements in targeted therapies or changes in screening guidelines, could impact the accuracy of our predictions. These potential shifts in medical practice could either mitigate or exacerbate the trends we have identified.

One of the major strengths of this study lies in its innovative approach to predicting future trends in CRC mortality rates using machine learning techniques, specifically the ARIMA model. This adds a new layer of depth to the existing body of research, which has primarily focused on epidemiological and biological aspects of CRC. Our study aligns with the well‐studied risk factors for early‐onset CRC and extends the current understanding by providing actionable predictive models. These models can be a valuable tool for healthcare policymakers and clinicians in planning targeted interventions. Additionally, our study is one of the few that specifically aims at predicting early‐onset CRC, filling a significant gap in the literature. The gender‐specific findings further add granularity to the study, enabling the development of gender‐focused preventive and therapeutic strategies.

This study has certain limitations. Being retrospective, it doesn't allow for causal inference. The database doesn't allow for adjustments based on socioeconomic status, race, and comorbidities, which could be potential confounding factors. The predictive models assume that future trends will mirror historical patterns, which may not account for potential changes in CRC screening, prevention, and treatment. While CDC WONDER database provides a comprehensive overview of mortality rates, it lacks granularity in demographic and clinical information. Additionally, there is concern of misrepresentation of cause of death.

Nevertheless, our projection based on 2015–2022 data demonstrated a similar trend in AAMR, albeit with a higher statistical error factor (Appendix S1). While the Ljung‐Box test suggests a good fit of the model, some level prediction errors (RMSE) are present, which are native to ARIMA models to indicate a measure of potential inaccuracies in precision forecasts. However, the overall trend would most likely remain the same, highlighting the importance of our study.

In conclusion, the increasing trend in CRC mortality highlights the necessity for more focused efforts in prevention, treatment, and compliance with guideline‐directed screening modalities. The gender disparities identified in our study warrant further investigation to understand their underlying causes and develop effective mitigation strategies. Finally, the prediction data suggests that an immediate call to action is needed to improve current efforts in CRC screening to shift the overall trend of CRC downward by 2035. The novelty and applicability of our results lie in their potential to guide future public health interventions and policy decisions, thereby contributing significantly to the existing body of knowledge.

## AUTHOR CONTRIBUTIONS

**Hassam Ali:** Conceptualization (equal); data curation (equal); formal analysis (equal); writing – original draft (equal); writing – review and editing (equal). **Pratik Patel:** Validation (equal); visualization (equal); writing – original draft (equal). **Dushyant Singh Dahiya:** Writing – original draft (equal); writing – review and editing (equal). **Manesh Kumar Gangwani:** Conceptualization (equal); writing – original draft (equal); writing – review and editing (equal). **Debargha Basuli:** Supervision (equal); validation (equal). **Babu Pappu Mohan:** Investigation (equal); supervision (equal); writing – review and editing (equal).

## CONFLICT OF INTEREST STATEMENT

The authors certify that they have no affiliations with or involvement in any organization or entity with any financial interest. The authors declare that they have no conflicts of interest.

## ETHICS STATEMENT

Institutional IRB approval was not obtained for this study as the CDC Wonder database is a third‐party de‐identified retrospective de‐identified database that is publicly accessible.

## PATIENT CONSENT STATEMENT

Not required due to retrospective de‐identified data analysis.

## Supporting information


Appendix S1.

## Data Availability

The datasets used and analyzed during the current study are freely available from the wide‐ranging online data for epidemiologic research database (https://wonder.cdc.gov/).
