# Supplementary material for: Prediction of early‐onset colorectal cancer mortality rates in the United States using machine learning
Source: Cancer Med. 2023 Dec 27;13(1):e6880. doi: 10.1002/cam4.6880 (PMC10807634; doi:10.1002/cam4.6880)
Supplement: Supplementary file 1 — Appendix S1. [file CAM4-13-e6880-s001.docx]

Supplementary Table 1: Total CRC-related mortality trends from 1999-2022

| Year | Deaths | Population | Age Adjusted Rate | Age Adjusted Rate Lower 95% Confidence Interval | Age Adjusted Rate Upper 95% Confidence Interval | Age Adjusted Rate Standard Error | % of Total Deaths |
| --- | --- | --- | --- | --- | --- | --- | --- |
| 1999 | 5549 | 121832902 | 4.7 | 4.6 | 4.8 | 0.1 | 4.00% |
| 2000 | 5726 | 122718203 | 4.7 | 4.6 | 4.9 | 0.1 | 4.20% |
| 2001 | 6126 | 123909542 | 4.9 | 4.8 | 5 | 0.1 | 4.50% |
| 2002 | 6106 | 123982489 | 4.8 | 4.7 | 5 | 0.1 | 4.40% |
| 2003 | 5876 | 124217955 | 4.6 | 4.5 | 4.7 | 0.1 | 4.30% |
| 2004 | 5685 | 124696761 | 4.4 | 4.3 | 4.5 | 0.1 | 4.10% |
| 2005 | 5754 | 125260089 | 4.4 | 4.3 | 4.5 | 0.1 | 4.20% |
| 2006 | 5993 | 125925139 | 4.6 | 4.4 | 4.7 | 0.1 | 4.40% |
| 2007 | 6201 | 126449632 | 4.6 | 4.5 | 4.8 | 0.1 | 4.50% |
| 2008 | 6347 | 126860406 | 4.8 | 4.6 | 4.9 | 0.1 | 4.60% |
| 2009 | 6314 | 127078241 | 4.7 | 4.6 | 4.8 | 0.1 | 4.60% |
| 2010 | 6499 | 127141270 | 4.8 | 4.7 | 5 | 0.1 | 4.70% |
| 2011 | 6445 | 127136655 | 4.8 | 4.7 | 4.9 | 0.1 | 4.70% |
| 2012 | 6494 | 127094479 | 4.8 | 4.7 | 5 | 0.1 | 4.70% |
| 2013 | 6594 | 127064809 | 5 | 4.9 | 5.1 | 0.1 | 4.80% |
| 2014 | 6541 | 127488488 | 5 | 4.9 | 5.1 | 0.1 | 4.80% |
| 2015 | 6542 | 127915146 | 5 | 4.9 | 5.1 | 0.1 | 4.80% |
| 2016 | 6520 | 127934078 | 5 | 4.9 | 5.1 | 0.1 | 4.70% |
| 2017 | 6582 | 128592994 | 5.1 | 5 | 5.2 | 0.1 | 4.80% |
| 2018 | 6600 | 128607361 | 5.1 | 5 | 5.3 | 0.1 | 4.80% |
| 2019 | 6433 | 128474367 | 5.1 | 4.9 | 5.2 | 0.1 | 4.70% |
| 2020 | 6529 | 128571971 | 5.2 | 5.1 | 5.3 | 0.1 | 4.70% |
| 2021 | 6633 | 129587395 | 5.2 | 5.1 | 5.3 | 0.1 | 4.93% |
| 2022 | 6819 | 129587395 | 5.3 | 5.2 | 5.5 | 0.1 | 5.7% |

Supplementary Table 2: CRC-related mortality trends from 1999-2022, Stratified by gender

| Gender | Year | Deaths | Population | Age Adjusted Rate | Age Adjusted Rate Lower 95% Confidence Interval | Age Adjusted Rate Upper 95% Confidence Interval | Age Adjusted Rate Standard Error | % of Total Deaths |
| --- | --- | --- | --- | --- | --- | --- | --- | --- |
| Female | 1999 | 2524 | 61246999 | 4.2 | 4 | 4.4 | 0.1 | 1.80% |
| Female | 2000 | 2526 | 61652646 | 4.1 | 4 | 4.3 | 0.1 | 1.80% |
| Female | 2001 | 2753 | 62256553 | 4.4 | 4.2 | 4.5 | 0.1 | 2.00% |
| Female | 2002 | 2681 | 62315837 | 4.2 | 4 | 4.3 | 0.1 | 2.00% |
| Female | 2003 | 2580 | 62484544 | 4 | 3.8 | 4.1 | 0.1 | 1.90% |
| Female | 2004 | 2493 | 62720088 | 3.8 | 3.7 | 4 | 0.1 | 1.80% |
| Female | 2005 | 2592 | 63027570 | 3.9 | 3.8 | 4.1 | 0.1 | 1.90% |
| Female | 2006 | 2725 | 63365526 | 4.1 | 3.9 | 4.2 | 0.1 | 2.00% |
| Female | 2007 | 2755 | 63628557 | 4.1 | 3.9 | 4.2 | 0.1 | 2.00% |
| Female | 2008 | 2769 | 63812680 | 4.1 | 3.9 | 4.2 | 0.1 | 2.00% |
| Female | 2009 | 2798 | 63906894 | 4.1 | 4 | 4.3 | 0.1 | 2.00% |
| Female | 2010 | 2828 | 63930821 | 4.1 | 3.9 | 4.2 | 0.1 | 2.10% |
| Female | 2011 | 2788 | 63849775 | 4.1 | 4 | 4.3 | 0.1 | 2.00% |
| Female | 2012 | 2838 | 63775210 | 4.2 | 4 | 4.4 | 0.1 | 2.10% |
| Female | 2013 | 2810 | 63708973 | 4.2 | 4 | 4.4 | 0.1 | 2.00% |
| Female | 2014 | 2798 | 63934001 | 4.2 | 4.1 | 4.4 | 0.1 | 2.00% |
| Female | 2015 | 2865 | 64113655 | 4.3 | 4.2 | 4.5 | 0.1 | 2.10% |
| Female | 2016 | 2809 | 64075438 | 4.3 | 4.2 | 4.5 | 0.1 | 2.00% |
| Female | 2017 | 2833 | 64326176 | 4.3 | 4.2 | 4.5 | 0.1 | 2.10% |
| Female | 2018 | 2759 | 64267850 | 4.3 | 4.1 | 4.4 | 0.1 | 2.00% |
| Female | 2019 | 2690 | 64151141 | 4.2 | 4.1 | 4.4 | 0.1 | 2.00% |
| Female | 2020 | 2752 | 64157032 | 4.3 | 4.1 | 4.5 | 0.1 | 2.00% |
| Female | 2021 | 2824 | 64364461 | 4.4 | 4.3 | 4.6 | 0.1 | 2.1% |
| Female | 2022 | 2917 | 64364461 | 4.6 | 4.4 | 4.8 | 0.1 | 2.17% |
| Male | 1999 | 3025 | 60585903 | 5.2 | 5 | 5.4 | 0.1 | 2.20% |
| Male | 2000 | 3200 | 61065557 | 5.4 | 5.2 | 5.5 | 0.1 | 2.30% |
| Male | 2001 | 3373 | 61652989 | 5.5 | 5.3 | 5.7 | 0.1 | 2.50% |
| Male | 2002 | 3425 | 61666652 | 5.5 | 5.3 | 5.7 | 0.1 | 2.50% |
| Male | 2003 | 3296 | 61733411 | 5.2 | 5.1 | 5.4 | 0.1 | 2.40% |
| Male | 2004 | 3192 | 61976673 | 5 | 4.8 | 5.2 | 0.1 | 2.30% |
| Male | 2005 | 3162 | 62232519 | 4.9 | 4.7 | 5.1 | 0.1 | 2.30% |
| Male | 2006 | 3268 | 62559613 | 5 | 4.8 | 5.1 | 0.1 | 2.40% |
| Male | 2007 | 3446 | 62821075 | 5.2 | 5 | 5.4 | 0.1 | 2.50% |
| Male | 2008 | 3578 | 63047726 | 5.4 | 5.2 | 5.6 | 0.1 | 2.60% |
| Male | 2009 | 3516 | 63171347 | 5.3 | 5.1 | 5.4 | 0.1 | 2.60% |
| Male | 2010 | 3671 | 63210449 | 5.5 | 5.3 | 5.7 | 0.1 | 2.70% |
| Male | 2011 | 3657 | 63286880 | 5.5 | 5.4 | 5.7 | 0.1 | 2.70% |
| Male | 2012 | 3656 | 63319269 | 5.6 | 5.4 | 5.7 | 0.1 | 2.70% |
| Male | 2013 | 3784 | 63355836 | 5.8 | 5.6 | 5.9 | 0.1 | 2.80% |
| Male | 2014 | 3743 | 63554487 | 5.8 | 5.6 | 5.9 | 0.1 | 2.70% |
| Male | 2015 | 3677 | 63801491 | 5.7 | 5.5 | 5.8 | 0.1 | 2.70% |
| Male | 2016 | 3711 | 63858640 | 5.8 | 5.6 | 5.9 | 0.1 | 2.70% |
| Male | 2017 | 3749 | 64266818 | 5.9 | 5.7 | 6 | 0.1 | 2.70% |
| Male | 2018 | 3841 | 64339511 | 6 | 5.8 | 6.2 | 0.1 | 2.80% |
| Male | 2019 | 3743 | 64323226 | 5.9 | 5.7 | 6.1 | 0.1 | 2.70% |
| Male | 2020 | 3777 | 64414939 | 6 | 5.9 | 6.2 | 0.1 | 2.70% |
| Male | 2021 | 3809 | 65222934 | 6 | 5.8 | 6.2 | 0.1 | 2.83% |
| Male | 2022 | 3902 | 65222934 | 6.1 | 5.9 | 6.3 | 0.1 | 2.9% |

Supplementary Table 3: Projected CRC-related mortality trends from 2023 to 2035, with 95% Confidence intervals

| Year | Age Adjusted Rate | Age Adjusted Rate Lower 95% Confidence Interval | Age Adjusted Rate Upper 95% Confidence Interval |
| --- | --- | --- | --- |
| 2023 | 5.3 | 5 | 5.6 |
| 2024 | 5.4 | 5 | 5.8 |
| 2025 | 5.4 | 5 | 5.9 |
| 2026 | 5.5 | 5 | 6 |
| 2027 | 5.6 | 5 | 6.1 |
| 2028 | 5.6 | 5.1 | 6.2 |
| 2029 | 5.7 | 5.1 | 6.3 |
| 2030 | 5.8 | 5.1 | 6.5 |
| 2031 | 5.9 | 5.2 | 6.6 |
| 2032 | 5.9 | 5.2 | 6.7 |
| 2033 | 6 | 5.3 | 6.8 |
| 2034 | 6.1 | 5.3 | 6.9 |
| 2035 | 6.2 | 5.4 | 7 |

Supplementary Table 4: Projected CRC-related mortality trends from 2023 to 2035, with 95% Confidence intervals, stratified by gender

|  | Females | | | Males | | |
| --- | --- | --- | --- | --- | --- | --- |
| Year | Age Adjusted Rate | Age Adjusted Rate Lower 95% Confidence Interval | Age Adjusted Rate Upper 95% Confidence Interval | Age Adjusted Rate | Age Adjusted Rate Lower 95% Confidence Interval | Age Adjusted Rate Upper 95% Confidence Interval |
| 2023 | 4.4 | 4.1 | 4.6 | 6.1 | 5.8 | 6.5 |
| 2024 | 4.4 | 4.1 | 4.7 | 6.2 | 5.8 | 6.7 |
| 2025 | 4.4 | 4.1 | 4.8 | 6.3 | 5.8 | 6.9 |
| 2026 | 4.5 | 4.1 | 4.9 | 6.4 | 5.8 | 7 |
| 2027 | 4.5 | 4.1 | 5 | 6.5 | 5.8 | 7.1 |
| 2028 | 4.6 | 4.1 | 5 | 6.6 | 5.9 | 7.3 |
| 2029 | 4.6 | 4.1 | 5.1 | 6.7 | 5.9 | 7.4 |
| 2030 | 4.7 | 4.1 | 5.2 | 6.8 | 6 | 7.6 |
| 2031 | 4.7 | 4.2 | 5.3 | 6.9 | 6 | 7.7 |
| 2032 | 4.8 | 4.2 | 5.3 | 7 | 6.1 | 7.9 |
| 2033 | 4.8 | 4.2 | 5.4 | 7.1 | 6.1 | 8 |
| 2034 | 4.9 | 4.3 | 5.5 | 7.2 | 6.2 | 8.2 |
| 2035 | 4.9 | 4.3 | 5.6 | 7.3 | 6.3 | 8.3 |

Supplementary Figure: Actual and projected rates of colorectal cancer-related mortality up to 2035, based on the ARIMA model from recent years (2015 to 2022); The shaded areas represent the 95% confidence intervals.


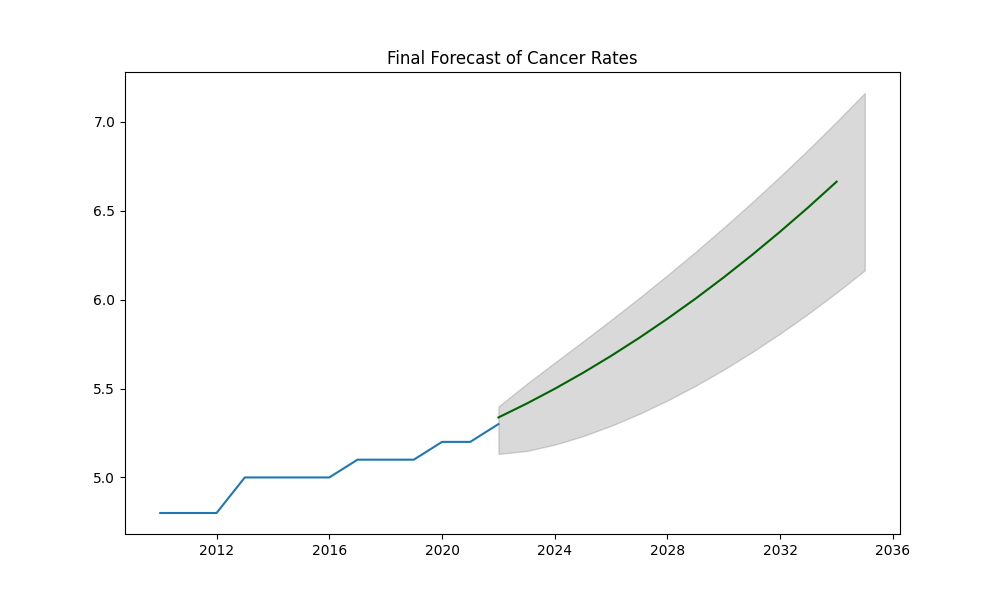


The forecasted rate for 2023 was 5.34/100,000 (95% CI: 5.15 - 5.53), with a projected increase to 6.66/100,000 (95% CI: 6.16 - 7.16) by 2035. The ARIMA model was (0, 1, 0) with a BIC of -26.47. The RMSE was 3.68.
